# Anti-inflammatory effect of rosiglitazone is not reflected in expression of NFκB-related genes in peripheral blood mononuclear cells of patients with type 2 diabetes mellitus

**DOI:** 10.1186/1472-6823-9-8

**Published:** 2009-02-25

**Authors:** Marjolijn CE Bragt, Jogchum Plat, Marco Mensink, Patrick Schrauwen, Ronald P Mensink

**Affiliations:** 1 Nutrigenomics Consortium, Top Institute Food and Nutrition, PO BOX 557, 6700 AN Wageningen, The Netherlands; 2 NUTRIM School for Nutrition, Toxicology and Metabolism, Department of Human Biology, Maastricht University Medical Centre+, PO Box 616, 6200 MD Maastricht, The Netherlands

## Abstract

**Background:**

Rosiglitazone not only improves insulin-sensitivity, but also exerts anti-inflammatory effects. We have now examined in type 2 diabetic patients if these effects are reflected by changes in mRNA expression in peripheral blood mononuclear cells (PBMCs) to see if these cells can be used to study these anti-inflammatory effects at the molecular level *in vivo*.

**Method:**

Eleven obese type 2 diabetic patients received rosiglitazone (2 × 4 mg/d) for 8 weeks. Fasting blood samples were obtained before and after treatment. Ten obese control subjects served as reference group. The expression of NFκB-related genes and PPARγ target genes in PBMCs, plasma TNFα, IL6, MCP1 and hsCRP concentrations were measured. In addition, blood samples were obtained after a hyperinsulinemic-euglycemic clamp.

**Results:**

Rosiglitazone reduced plasma MCP1 and hsCRP concentrations in diabetic patients (-9.5 ± 5.3 pg/mL, *p * = 0.043 and -1.1 ± 0.3 mg/L *p * = 0.003), respectively). For hsCRP, the concentration became comparable with the non-diabetic reference group. However, of the 84 NFκB-related genes that were measured in PBMCs from type 2 diabetic subjects, only RELA, SLC20A1, INFγ and IL1R1 changed significantly (*p * < 0.05). In addition, PPARγ and its target genes (CD36 and LPL) did not change. During the clamp, insulin reduced plasma MCP1 concentration in the diabetic and reference groups (-9.1 ± 1.8%, *p * = 0.001 and -11.1 ± 4.1%, *p * = 0.023, respectively) and increased IL6 concentration in the reference group only (23.5 ± 9.0%, *p * = 0.028).

**Conclusion:**

In type 2 diabetic patients, the anti-inflammatory effect of rosiglitazone is not reflected by changes in NFκB and PPARγ target genes in PBMCs *in vivo*. Furthermore, our results do not support that high insulin concentrations contribute to the pro-inflammatory profile in type 2 diabetic patients.

## Background

Rosiglitazone, an agonist for the nuclear receptor peroxisome proliferator-activated receptor gamma (PPARγ), is a widely used drug for the treatment of type 2 diabetes mellitus. It belongs to the group of thiazolidinediones (TZD) and increases insulin sensitivity of peripheral tissues. In addition, there is evidence that rosiglitazone has anti-inflammatory effects [1-3]. It has been postulated that rosiglitazone exerts its anti-inflammatory effect through inhibition of the transcription factor nuclear factor κB (NFκB) pathway [1]. Normally, NFκB is bound in the cytosol to its inhibitor κB (IκB) to prevent activation of NFκB. Inflammatory signals can cause phosphorylation of IκB, thereby releasing and activating NFκB, followed by translocation of NFκB to the nucleus and activation of genes involved in the pro-inflammatory response, such as TNFα and matrix metalloproteinases [4]. To further understand in humans the *in vivo * (anti-inflammatory) effects of rosiglitazone treatment at the molecular level, it would be advantageous that Peripheral Blood Mononuclear Cells (PBMCs) could be used. These cells are readily accessible in humans compared to the relatively inaccessible target tissues of PPARγ ligands, namely adipose tissue and liver [5]. It has already been demonstrated that monocytes respond to PPARγ ligands by reducing the expression of inflammatory cytokines *ex vivo*, such as TNFα, IL-6 and IL1b [6]. In addition, Mohanty *et al*. [3] have shown that rosiglitazone inhibits the binding of NFκB to DNA in the nucleus of PBMCs from obese and obese diabetic patients. This was accompanied by an anti-inflammatory effect, as indicated by reduced plasma hsCRP and MCP-1 concentrations [3]. However, it is not known if rosiglitazone's anti-inflammatory effect changes the transcription of NFκB related genes in PBMCs *in vivo*. Therefore, the first aim of our study was to examine if anti-inflammatory properties of rosiglitazone are reflected in changes in expression of genes involved in the NFκB signaling pathway in PBMCs. Furthermore, the role of an increased insulin concentration in low-grade systemic inflammation, as often seen in type 2 diabetic patients, is not completely understood [7]. Therefore, the second aim of this study was to examine plasma inflammatory markers in the fasted (hyperglycaemic) state and during hyperinsulinemia using a hyperinsulinemic-euglycemic clamp.

## Methods

### Subjects

Twelve middle-aged obese men with well-controlled type 2 diabetes mellitus participated in this study. Due to extreme changes in serum cholesterol concentrations and gene expression profiles, one subject was excluded from the statistical analyses. The characteristics of the 11 remaining (age, 62 ± 5 y; body mass index (BMI), 31 ± 3 kg/m^2^) type 2 diabetic subjects and 10 BMI and age matched normoglycemic controls (age, 57 ± 8 y; BMI, 30 ± 4 kg/m^2^) are shown in table 1. Type 2 diabetes mellitus was diagnosed at least 1 year before the study, and most patients were treated with sulphonylurea (n = 3) or metformin (n = 5) or both (n = 1) as anti-diabetic medication. Control subjects had normal glucose homeostasis, as determined with a standard oral glucose tolerance test, and had no family history of diabetes. The Medical Ethical Review Board of Maastricht University had approved the study and written, informed consent was obtained from all volunteers.

**Table 1:** BMI and plasma parameters of type 2 diabetic patients before and after rosiglitazone treatment and of non-diabetic controls.

	Diabetic patients (n = 11)		Controls (n = 10)
	Before treatment	After treatment	
BMI (kg/m^2^)	30 ± 1.0	31 ± 1.0	31 ± 1.3
Glucose (mmol/L)	9.2 ± 0.5^a^	8.1 ± 0.5^b,c^	5.8 ± 0.1
Insulin (mU/L)	24.1± 3.1^a^	17.2 ± 1.7^b,c^	12.3 ± 0.9
GIR (μmol/kg/min)	12.8± 1.3^a^	20.6 ± 1.7^b^	24.6 ± 2.4
Total cholesterol (mmol/L)	5.4 ± 0.3	5.7 ± 0.4	5.2 ± 0.3
LDL cholesterol (mmol/L)	3.5 ± 0.2	3.8 ± 0.4	3.4 ± 0.3
HDL cholesterol (mmol/L)	0.93 ± 0.1	1.10± 0.1^b^	1.14 ± 0.1
TCH/HDL ratio	6.9 ± 1.1	5.5 ± 1.0	5.5 ± 1.1
Triglycerides (mmol/L)	2.0 ± 0.3	1.5 ± 0.2^b^	1.5 ± 0.4
FFA (mmol/L)	509 ± 47	400 ± 37^b^	513 ± 45.6

^a ^Significantly different (*P * < 0.05) between diabetic patients and non-diabetic controls

^b ^Significant effect (*P * < 0.05) of rosiglitazone treatment in diabetic patients

^c ^Significantly different (*P * < 0.05) between diabetic patients after rosiglitazone treatment and non-diabetic controls

### Study design

Details of this study have been described previously [8,9]. In short, diabetic patients stopped any anti-diabetic medication 14 days before the start of the study. Subjects were asked not to participate in (exhaustive) physical activity the last three days preceeding the measurements, and to consume a diet according to the Dutch guidelines for a healthy diet. After a baseline hyperinsulinemic-euglycemic clamp, diabetic patients were treated with rosiglitazone (Avandia^®^, GlaxoSmithKline, Zeist, the Netherlands), 8 mg/day (2 × 4 mg) for 8 weeks. After rosiglitazone treatment, subjects underwent a second clamp. Blood samples were drawn before and after the clamp, thereby providing fasting and insulin-stimulated blood samples before and after rosiglitazone treatment.

The non-diabetic patients served as baseline controls to the diabetic patients. They also underwent a hyperinsulinemic-euglycemic clamp, but were not treated with rosiglitazone.

### Hyperinsulinemic-euglycemic clamp

After an overnight fast, subjects came to the laboratory at 8 AM. A cannula was inserted into each antecubital vein for the infusion of tracer, insulin and glucose. A third cannula was inserted retrogradely into a superficial dorsal hand vein for arterialized blood sampling. After taking fasting blood samples, a primed constant infusion of [6,6]-^2^H_2_-glucose was initiated (0.04 mg/kg·min) for 300 minutes. At t = 120 min, a 3-hour primed constant infusion of insulin (Actrapid, Novo Nordisk, Bagsvaerd, Denmark) was started (40 mU/m^2^·min), and glucose was clamped by a variable co-infusion of 20% glucose with tracer added. Blood samples taken at t = 0 min and t = 300 min were used for further analysis.

### Plasma measurements

EDTA plasma was obtained by 10 minutes of centrifugation at 3000 rpm immediately after sampling, and stored at -80°C for later analysis. Concentrations of total cholesterol (ABX Diagnostics, Montpelier, France), HDL cholesterol (precipitation method; Roche Diagnostics Corporation, Indianapolis, IN), and triglycerides corrected for free glycerol (Sigma-Aldrich Chemie, Steinheim, Germany) were analysed enzymatically. Serum LDL cholesterol concentrations were calculated by using the formula of Friedewald *et al*. [10]. Insulin concentrations were measured using a RIA (Linco Research. St. Charles, MO, USA). Free fatty acids (FFA) were determined using the Wako Nefa C test kit (Wako Chemicals, Neuss, Germany) and plasma glucose was determined by using the hexokinase method (LaRoche, Basel, Switzerland). The glucose infusion rate (GIR) was used as a measure of insulin sensitivity. For the inflammation markers, high sensitive CRP (hsCRP) was measured on Cobas Mira with a commercial available kit (Kamiya Biomedical Company, Seattle, WA, USA) and TNFα, IL6 and MCP1 were measured with an ELISA kit from R&D systems. All samples from one subject were analysed in the same analytical run. Samples were corrected for plasma albumin concentrations to correct for changes in blood volume due to fluid infusion during the hyperinsulinemic-euglycemic clamp [11].

### PBMC isolation and total RNA isolation

PBMCs were isolated from an EDTA anti-coagulated peripheral fasted blood sample of diabetic patients only, directly after blood sampling through gradient centrifugation using lymphoprep (Nycomed, Oslo, Norway) according to the instructions from the manufacturer. The obtained PBMCs were immediately lysed and homogenised in 1.5 ml Trizol (Invitrogen Life Technologies, Breda, The Netherlands) for RNA stabilisation and subsequent RNA isolation according a standardized protocol as described by the manufacturer. Next, RNA was purified using the RNeasy mini kit (Qiagen Benelux B.V., Venlo, the Netherlands) followed by dissolving the RNA in RNAse and DNAse free water (Invitrogen Life Technologies, Breda, The Netherlands). RNA purity was measured on the NanoDrop 1000 (NanoDrop Technologies, Wilmington, DE, USA), and considered suitable for further processing at 260/280 and 260/230 ratios of > 1.7. Integrity was evaluated using the BioAnalyzer (Agilent, Palo Alto CA, USA) and considered to be intact with an RNA integrity number > 7.0.

### Real time PCR

cDNA was synthesised using the ReactionReady™ First Strand cDNA Synthesis Kit (SuperArray Bioscience corporation, Frederick, MD, USA). The total amount of RNA used in the reaction varied from 800 ng to 1 μg. Equal amounts of RNA were used for samples before and after treatment of the same subject.

Gene expression of NFκB pathway focussed genes was analysed using NFκB RT^2 ^*Profiler * PCR arrays, according to the manufacturer's instructions (SuperArray Bioscience Corporation, Frederick, MD, USA). The PCR array consisted of a 96-well plate including primer sets of 84 NFκB related genes (see additional file 1), plus 5 housekeeping genes and 2 negative controls. The primer sets were optimised for real time detection using RT^2 ^Real Time™ SYBR Green PCR master mix (SuperArray Bioscience Corporation, Frederick, MD, USA). In addition, the relative expression levels of PPARγ, CD36 and LPL, were also determined with real time PCR, using assays-on-demand and Taqman Universal Mastermix (Applied Biosystems, Foster City, CA, USA). All gene expression analyses were performed on the ABI PISM 7000 system (Applied Biosystems, Foster City, CA, USA).

### Data analysis of real time PCR data

The No Template Control tested for DNA contamination in the PCR system and the No Reverse Transcription Control tested for contamination of the original RNA with genomic DNA. Those threshold cycles were above 35, which indicates that there was no contamination. The average C_t _values of the housekeeping genes, β-actin and 18srRNA, were used for normalisation of each individual sample. To express the difference in expression before and after rosiglitazone treatment in diabetic patients, the ΔΔC_t _value was calculated for each gene according to the comparative Ct method, and was used for statistical analysis.

### Statistics

Fasting plasma parameters are presented as mean ± SEM. Gene expression levels were not normally distributed and are therefore expressed as median ΔΔC_t _values together with corresponding ranges. An independent Student's t-test was used to compare diabetic patients before or after rosiglitazone with control subjects. Results of plasma parameters before and after rosiglitazone treatment in type 2 diabetic patients were analysed using a paired Student's t-test. The effect of rosiglitazone on gene expression levels in type 2 diabetic patients was analysed with the non-parametric Wilcoxon signed rank test. Changes (%) in plasma parameters during the hyperinsulinemic-euglycemic clamp (T0 vs. T300) were tested with a one sample t-test. In addition, using the Student's t-test, the changes in plasma parameters induced by the hyperinsulinemic-euglycemic clamp before rosiglitazone treatment were compared to the clamp-induced changes after rosiglitazone treatment.

All statistical analyses were performed with SPSS 14.0 for Windows (SPSS Inc., Chicago, IL, USA).

## Results

### Body weight

There was no difference in body weight between diabetic patients and the BMI-matched controls (94.3 ± 4.1 kg and 92.7 ± 4.1 kg, respectively (*p * = 0.778)). Rosiglitazone treatment resulted in a slight, but non-significant increase in body weight during the 8 weeks follow-up period in the diabetic patients (1.3 ± 1.0 kg, *p * = 0.21).

### Plasma glucose and insulin concentrations and insulin sensitivity

As expected, diabetic patients were less insulin-sensitive and had higher fasting plasma glucose and insulin concentrations compared with non-diabetic controls (Table 1). Fasting glucose and insulin were significantly decreased in the diabetic patients after rosiglitazone treatment (-1.0 ± 0.5 mmol/L, *p * = 0.044) and (-6.9 ± 2.3 mU/L, *p * = 0.013, respectively), while insulin sensitivity increased (change in GIR: +7.8 ± 1.9 μmol/kg/min, *p * = 0.002) (Table 1).

### Plasma lipid concentrations

As shown in Table 1, there were no significant differences in plasma total cholesterol, LDL cholesterol, HDL cholesterol, TCH/HDL ratio, triglycerides and free fatty acids (FFA) between diabetic patients and non-diabetic controls. Fasting HDL cholesterol increased significantly (+0.21 ± 0.07 mmol/L, *p * = 0.013), while triglyceride and FFA concentrations decreased (-0.48 ± 0.21 mmol/L, *p * = 0.042 and -109 ± 38 mmol/L, *p * = 0.018 respectively) upon rosiglitazone treatment in diabetic patients. Total cholesterol and LDL cholesterol concentrations were not significantly affected by rosiglitazone treatment (+0.30 ± 0.20 mmol/L, *p * = 0.152 and +0.30 ± 0.20 mmol/L, *p * = 0.160, respectively). The TCH/HDL ratio tended to improve after treatment (-1.3 ± 0.7), but this did not reach statistically significance (*p * = 0.09).

### Plasma hsCRP, TNFα, IL6 and MCP1 concentrations

**Figure 1:**
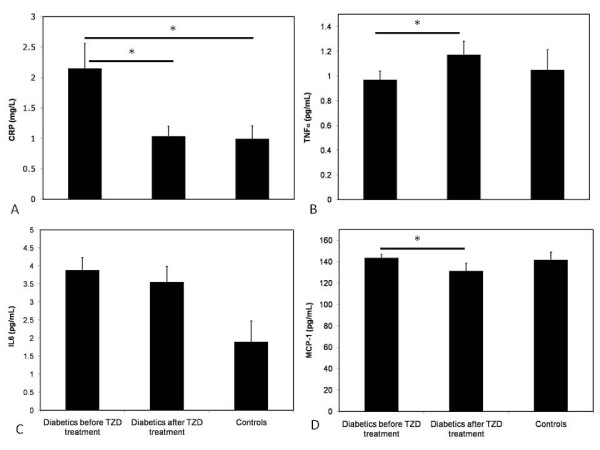
**Concentrations of plasma inflammation markers in controls and in diabetics before and after rosiglitazone treatment**. Baseline concentrations of plasma inflammatory markers, A) hsCRP, B) TNFα, C) IL6 and D) MCP1, in type 2 diabetic and control subjects and levels after rosiglitazone treatment in type 2 diabetic patients. * = *p-value *< 0.05.

As shown in figure 1, the basal plasma concentration of hsCRP in the diabetic patients was 2.2 ± 0.4 mg/L, which was significantly higher than that of the non-diabetic controls (1.0 ± 0.2 mg/L, *p * = 0.027). There were no differences in TNFα, IL6 and MCP1 concentrations between diabetic patients and control subjects. As a result of rosiglitazone treatment, basal hsCRP and MCP1 concentrations significantly decreased (-1.1 ± 0.3 mg/L, *p * = 0.003 and -9.5 ± 5.3 pg/mL, *p * = 0.043, respectively) and, paradoxically, TNFα concentrations increased (+0.22 ± 0.1 pg/mL, *p * = 0.037) in type 2 diabetic patients. After rosiglitazone treatment, hsCRP (1.0 ± 0.16 mg/L) and MCP1 concentrations (132 ± 7 pg/mL) in diabetic patients were comparable with those measured in non-diabetic controls.

### Expression of PPARγ, PPARγ responsive genes and NFκB related genes

Of the 84 genes measured, 75 were detectable in PBMCs and only the expression of genes encoding for IFNγ (interferon gamma), IL1R1 (interleukin 1 receptor 1), RELB (V-rel reticuloendotheliosis viral oncogene homolog B, nuclear factor of kappa light polypeptide gene enhancer in B-cells 3 (avian)) and SLC20A1 (solute carrier family 20 (phosphate transporter) member 1) changed significantly in the diabetic patients upon rosiglitazone treatment (table 2).

**Table 2:** Effect of rosiglitazone treatment on expression levels (ΔC_t_) of PPARγ, PPARγ responsive genes and NFκB related genes in type 2 diabetic patients^1,2^

Gene	ΔΔC_t _(after – before treatment)	*p*-value^3^
PPARγ	0.31 (-0.70 – 1.29)	0.213
CD36	0.37 (-0.93 – 0.90)	0.248
LPL	-0.39 (-0.90 – 1.26)	0.248
INFγ	-0.53 (-3.04 – 0.62)	0.045
IL1R1	0.37 (-1.16 – 1.51)	0.041
RELB	0.31 (-0.23 – 2.27)	0.016
SLC20A1	0.41 (-1.03 – 1.93)	0.033

1 The expression of 84 genes related to NFκB was profiles using the RT^2^Profiler™ PCR Array; n = 11

2 Values are reported as ΔΔC_t _values and presented as medians with ranges. A negative ΔΔC_t _value corresponds with an upregulation and a positive ΔΔC_t _value with a downregulation of the gene after rosiglitazone treatment.

3 Gene expression levels (ΔC_t _values) were analysed with the non-parametric Wilcoxon signed rank test. Differences were considered significant at a *p*-value < 0.05

The expression level of PPARγ itself was not changed by its ligand rosiglitazone. Also expression levels of CD36 (thrombospondin receptor) and LPL (lipoprotein lipase), which are known PPARγ target genes [12-14], did not change (Table 2).

### Changes in plasma parameters during the clamp

Before rosiglitazone treatment, plasma albumin concentrations were 38.1 ± 0.6 g/L before the clamp and 36.8 ± 0.5 g/L after the clamp (*p * = 0.005). These concentrations were comparable after rosiglitazone treatment. For the control subjects, these values were before and after the clamp respectively 38.7 ± 0.6 g/L and 36.4 ± 0.7 g/L (*p * < 0.001). The observed reductions in plasma albumin concentration indicate that due to the glucose infusion, the plasma volume had changed during the clamp. Therefore, levels of the plasma parameters were related to those of albumin. It is known that plasma albumin concentrations are stable during the day [11].

Before treatment, serum total, LDL, and HDL cholesterol levels did not change in response to insulin during the hyperinsulinemic-euglycemic clamp in both type 2 diabetic patients and control subjects (Table 3). Triglycerides levels decreased in control subjects (-16.7 ± 4,5%, *p * = 0.007) and also in diabetic patients after rosiglitazone treatment (-14.3 ± 5.5%, *p * = 0.027). FFA levels decreased significantly in type 2 diabetic patients (-58.9 ± 3.4%, *p * < 0.001) in response to insulin. In control subjects the reduction in FFA was even more pronounced (-80.1 ± 2.6%, *p * < 0.001). Rosiglitazone improved the insulin-induced decrease in FFA (from -58.9 ± 3.4% to -65.0 ± 4.1%, *p * = 0.045).

**Table 3:** Relative changes in plasma parameters upon insulin-stimulation during the hyperinsulinemic-euglycemic clamp

	Diabetic patients (n = 11)		Controls (n = 10)
	Before treatment	After treatment	
Albumin (%)	-3.4 ± 0.9^#^	-3.1 ± 1.1^#^	-6.0 ± 0.8^#^
Total cholesterol (%)	0.4 ± 0.8	-1.7 ± 1.4	0.4 ± 0.7
LDL cholesterol (%)	2.5 ± 2.1	-0.5 ± 1.3	2.3 ± 1.1
HDL cholesterol (%)	1.3 ± 2.7	0.8 ± 1.6	2.7 ± 1.3
Triglycerides (%)	-5.2 ± 6.4	-14.3 ± 5.5^a,c^	-16.7 ± 4.5^a^
FFA (%)	-58.9 ± 3.4^a,b^	-65.0 ± 4.1^a,c^	-80.1 ± 2.6^a^
hsCRP (%)	-4.7 ± 2.7	-4.9 ± 2.4	5.5 ± 4.5
TNFα (%)	12.0 ± 6.6	-9.1 ± 7.9^c^	12.1 ± 14.2
IL6 (%)	13.7 ± 6.7	16.4 ± 7.9	23.5 ± 9.0^a^
MCP1 (%)	-9.1 ± 1.8^a^	-3.7 ± 4.0	-11.1 ± 4.1^a^

^# ^Plasma albumin changed (p < 0.05) during the clamp because of increase in blood volume due to fluid infusion. Therefore, all plasma parameters are corrected for changes in plasma albumin concentration and presented as mean ± SEM

^a ^Significant change (*P * < 0.05) in response to insulin

^b ^Significant difference between type 2 diabetic patients and BMI-matched controls in response to insulin

^c ^Significant change (*P * < 0.05) effect of rosiglitazone treatment on the response to insulin in type 2 diabetic patients

Insulin significantly decreased MCP1 levels in type 2 diabetic patients before rosiglitazone treatment (-9.1 ± 1.8%, *p * = 0.001) and control subjects (-11.1 ± 4.1%, *p * = 0.023) (table 3). Control subjects also showed a significant increase in IL6 levels (23.5 ± 9.0%, *p * = 0.028) Changes in TNFα levels upon insulin stimulation differed significantly before and after rosiglitazone treatment in diabetic patients. Levels of TNFα increased during the clamp before rosiglitazone treatment (+12.0 ± 6.6%), but decreased after rosiglitazone treatment (-9.1 ± 7.9%, *p * = 0.006).

## Discussion

In this study, 8 weeks of rosiglitazone treatment (2 × 4 mg/d) not only improved insulin sensitivity and plasma lipids, but also lowered fasting plasma concentrations of hsCRP and MCP1. After rosiglitazone, fasting concentrations of hsCRP and MCP1 in diabetic patients were even comparable to those of non-diabetic controls. These anti-inflammatory changes were not reflected in the expression of NFκB-related genes in PBMCs.

Reductions in fasting triglyceride concentrations have been reported for other TZDs, such as pioglitazone, troglitazone and darglitazone. We have now shown that these results can be extended to rosiglitazone. Furthermore, FFA concentrations were also reduced after rosiglitazone treatment. These reductions may be explained by an increased clearance of TG and FFA, as shown by Dhindsa *et al*. [15]. Rosiglitazone treatment further significantly increased serum HDL cholesterol concentrations and total cholesterol and LDL cholesterol tended to increase Generally, the effects of rosiglitazone on serum lipid concentrations were comparable to those of other TZDs [16].

Rosiglitazone treatment in type 2 diabetic patients decreased hsCRP and MCP1 concentrations, indicating a reduced inflammatory state. Mohanty *et al*. also observed a significant reduction in MCP1 and hsCRP concentrations after 6 weeks of rosiglitazone treatment (4 mg/d) in non-diabetic obese subjects and obese diabetic patients [3]. In accordance with previous studies [2,17,18], we did not observe an effect of rosiglitazone on plasma IL6 concentrations. The increase we observed in TNFα concentrations is unexpected, although studies on the effects of TZDs on plasma TNFα concentrations are inconsistent. Some studies have shown that plasma concentrations of this cytokine are increased in subjects with insulin resistance or type 2 diabetes [19,20]. A number of other studies, however, showed reduced TNFα concentrations [21,22] after rosiglitazone treatment in type 2 diabetic patients, whereas others found no effect [18]. In line with our observations, Goldstein *et al*. [23] showed that TNFα concentrations were significantly increased when rosiglitazone was added to metformin treatment in type 2 diabetic patients. Due to these inconsistent results, a clear explanation is lacking.

Despite the rosiglitazone-induced reductions in fasting plasma CRP and MCP1, which are under control of NFκB [24,25], expression of NFκB-related genes in PBMCs hardly changed upon rosiglitazone treatment. Of the 75 detectable NFκB-related genes in PBMCs, only 4 genes were significantly altered. The gene encoding for interferon gamma (INFγ) was significantly upregulated, whereas ILR1, RELB and SLC20A1 were significantly downregulated by rosiglitazone treatment. These changes would indicate an inhibition of the NFκB signalling cascade [4,26-28]. The significant reduction in MCP1 and hsCRP concentrations after 6 weeks of rosiglitazone treatment (4 mg/d) in non-diabetic obese subjects and obese diabetic patients as described by Mohanty *et al*., was accompanied by a significant fall in intranuclear NFκB levels in PBMCs [3]. This suggests a direct interference of PPARγ in the binding of NFκB to the promotor regions of proinflammatory genes. However, these reduced intranuclear NFκB levels were not accompanied by a changed expression of the NFκB-related genes IkB and p65 (REL A) after rosiglitazone treatment [3,29]. For troglitazone, another PPARγ agonist, it has also been demonstrated that intranuclear and cellular levels of NFκB were decreased in mononuclear cells of obese subjects [30]. Unfortunately, these studies did not examine if the fall in intranuclear NFκB levels was accompanied by a reduced expression of inflammatory NFκB target genes. In our study, the lack of effect on NFκB related genes by rosiglitazone in PBMCs suggests that PBMCs are insensitive to rosiglitazone.

To further substantiate whether PBMCs were non-responsive to rosiglitazone treatment, we evaluated if rosiglitazone influenced gene expression of specific PPARγ dependent genes in PBMCs *in vivo*, i.e., PPARγ itself, CD36 and lipoprotein lipase (LPL). *In vitro * and *ex vivo * studies have shown that expression of CD36 [31,32] and LPL [33] in macrophages increased after activation of PPARγ. In our study, however, the *in vivo * expression of PPARγ, CD36 and LPL in PBMCs was not altered by rosiglitazone treatment, which suggests that PBMCs are non-responsive to rosiglitazone. In contrast to most other studies, we used PBMCs, a mixed population of white blood cells, and studied the *in vivo * effects. Most studies that did observe anti-inflammatory effects of rosiglitazone focussed on *in vitro * effects in a specific subpopulation of blood cells, namely monocytes or macrophages. These studies showed an increased expression of PPARγ and PPARγ responsive genes when these cells were cultured *ex vivo * or *in vitro*, and/or were given extra cytokine stimulation [31,34,35]. *In vivo*, Bouhlel *et al*. [34] showed a significant upregulation of PPARγ expression in PBMCs of subjects after 2 months of 45 mg/day pioglitazone (TZD) treatment in patients with severe atherosclerosis, although expression of the PPARγ dependent genes, CD136 and mannose receptor, did not change. Combining these finding with our results suggests that gene expression profiles in PBMCs are not suitable to use as a biomarker to study *in vivo * the effects of rosiglitazone intervention at a molecular level. Whether specific subpopulations of cells within PBMCs are suitable, warrant further investigation. In this respect, study of monocytes, preferably isolated from blood via for example cell sorter techniques, warrants attention as especially the monocytes play an important role in the inflammation process and insulin resistance. Arkan *et al*. [36], for example, showed that deletion of IKKβ, required for the activation of NFκB, in myeloid precursor cells protected animals from diet-induced obesity-related insulin resistance.

To investigate the acute effect of insulin on the low-grade pro-inflammatory profile in type 2 diabetic patients at constant glucose concentrations, plasma inflammation parameters were analysed before and after the hyperinsulinemic-euglycemic clamp. Results, however, were not conclusive. MCP1 level decreased in both diabetic and control subjects, IL6 level increased only in control subjects, while hsCRP and TNFα levels did not significantly change. The reduced MCP1 level after insulin stimulation can, however, also be an indirect effect of insulin and explained for example by the insulin-mediated reduction in FFA [37]. The causal role of IL6 in the development of insulin resistance and type 2 diabetes is not clear. Infusion with a physiological concentration of IL6 in humans increased the uptake of glucose in subcutaneous adipose tissue *in vivo *[38]. On the other hand, IL6 levels are increased in type 2 diabetic subjects. Like in our study, Krogh-Madsen *et al*. found an increase in plasma IL6 concentrations during a hyperinsulinemic-euglycemic clamp in healthy human males [39]. This increase was accompanied by an elevated insulin-stimulated increase in IL6 gene expression in adipose tissue. Also, they found no change in plasma concentrations of TNFα, which expression was increased in adipose tissue and reduced in muscle tissue [39]. The reason that we did not find a reduction in hsCRP concentration may have been due to the relative short time of insulin infusion in our study, as previous studies demonstrated decreased hsCRP concentrations after 10 or more hours of insulin infusion [40-42]. It also needs to be mentioned that during the hyperinsulinemic-euglycemic clamp, not only insulin infusion can exerts biological effects, but also the amount of glucose infused to maintain euglycemia [43]. However, Dandona *et al*. have demonstrated an acute anti-inflammatory effect of insulin infusion at a physiological concentration, infusing insulin (2.0–2.5 IU/h) – dextrose(5%) and in obese non-diabetic subjects, while maintaining glucose levels as close to basal levels as possible [41]. This anti-inflammatory effect was reflected by reduced intranuclear levels of NFκB, increased IκB, decreased ROS generation and decreased p47phox subunit in PBMCs, decreased plasma intracellular adhesion molecule 1, plasminogen inhibitor 1 and MCP1 concentrations [41]. Infusion with only dextrose or saline had no effects on these parameters [41]. Taken together, a supra-physiological increase in insulin concentration does not per se contribute to the disturbances in inflammatory markers in type 2 diabetic patients.

## Conclusion

In conclusion, 8 weeks of rosiglitazone treatment (2 × 4 mg/d) resulted in improved insulin sensitivity and lipid profile and reduced concentrations of plasma inflammatory markers (MCP1 and hsCRP) in type 2 diabetic patients. Furthermore, plasma inflammatory parameters did not change consistently during the clamp in both diabetic and control patients, which does not suggest that high insulin levels contribute to the proinflammatory state in type 2 diabetic patients. Finally, the anti-inflammatory effect of rosiglitazone is not reflected by changes in NFκB and PPARγ target genes in PBMCs *in vivo*.

## Competing interests

The authors declare that they have no competing interests.

## Authors' contributions

All authors contributed to the design, execution, and analysis of this study and writing the manuscript. All authors read and approved the final manuscript.

## Pre-publication history

The pre-publication history for this paper can be accessed here:



## Supplementary Material

Additional file 1 **84 NFκB-related genes measured with the NFκB RT^2 ^*Profiler * PCR array.** The gene table provided represents the 84 NFκB-related genes measured with the NFκB RT^2 ^*Profiler * PCR array Click here for file
