# Supplementary material for: Anti-inflammatory effect of rosiglitazone is not reflected in expression of NFκB-related genes in peripheral blood mononuclear cells of patients with type 2 diabetes mellitus
Source: BMC Endocr Disord. 2009 Feb 25;9:8. doi: 10.1186/1472-6823-9-8 (PMC2653037; doi:10.1186/1472-6823-9-8)
Supplement: Additional file 1 — 84 NFκB-related genes measured with the NFκB RT2 Profiler PCR array. The gene table provided represents the 84 NFκB-related genes measured with the NFκB RT2 Profiler PCR array [file 1472-6823-9-8-S1.pdf]

Additional file 1 - NFκB-related genes measured with the NFκB RT[2]*Profiler* PCR array

Gene name	Gene function	Unigene code
AGT[#]	Angiotensinogen (serpin peptidase inhibitor, clade A, member 8)	Hs.19383
AKT1	V-akt murine thymoma viral oncogene homolog 1	Hs.525622
ATF1	Activating transcription factor 1	Hs.435267
BCL10	B-cell CLL/lymphoma 10	Hs.567382
BCL3	B-cell CLL/lymphoma 3	Hs.31210
BF	B-factor, properdin	Hs.69771
BIRC2	Baculoviral IAP repeat-containing 2	Hs.503704
CARD4	Caspase recruitment domain family, member 4	Hs.405153
CASP1	Caspase 1, apoptosis-related cysteine peptidase (interleukin 1, beta, convertase)	Hs.2490
CASP8	Caspase 8, apoptosis-related cysteine peptidase	Hs.369736
CCL2	Chemokine (C-C motif) ligand 2	Hs.303649
CD40	CD40 antigen (TNF receptor superfamily member 5)	Hs.472860
CFLAR	CASP8 and FADD-like apoptosis regulator	Hs.390736
CHUK	Conserved helix-loop-helix ubiquitous kinase	Hs.198998
CSF2[#]	Colony stimulating factor 2 (granulocyte-macrophage)	Hs.1349
CSF3[#]	Colony stimulating factor 3 (granulocyte)	Hs.2233
SLC44A2	Solute carrier family 44, member 2	Hs.515134
EDARADD	EDAR-associated death domain	Hs.352224
EDG2	Endothelial differentiation, lysophosphatidic acid G-protein-	Hs.126667

	coupled receptor, 2	
EGR1#	Early growth response 1	Hs.326035
ELK1#	ELK1, member of ETS oncogene family	Hs.181128
F2R	Coagulation factor II (thrombin) receptor	Hs.482562
FADD	Fas (TNFRSF6)-associated via death domain	Hs.86131
FASLG	Fas ligand (TNF superfamily, member 6)	Hs.2007
FOS	V-fos FBJ murine osteosarcoma viral oncogene homolog	Hs.25647
GJA1	Gap junction protein, alpha 1, 43kDa (connexin 43)	Hs.74471
HMOX1	Heme oxygenase (decycling) 1	Hs.517581
HTR2B	5-hydroxytryptamine (serotonin) receptor 2B	Hs.421649
ICAM1	Intercellular adhesion molecule 1 (CD54), human rhinovirus receptor	Hs.515126
IFNA1#	Interferon, alpha 1	Hs.37026
IFNB1	Interferon, beta 1, fibroblast	Hs.93177
IFNG	Interferon, gamma	Hs.856
IKBKB	Inhibitor of kappa light polypeptide gene enhancer in B-cells, kinase beta	Hs.413513
IKBKE	Inhibitor of kappa light polypeptide gene enhancer in B-cells, kinase epsilon	Hs.321045
IKBKG	Inhibitor of kappa light polypeptide gene enhancer in B-cells, kinase gamma	Hs.43505
IL10#	Interleukin 10	Hs.193717
IL1A#	Interleukin 1, alpha	Hs.1722
IL1B	Interleukin 1, beta	Hs.126256

IL1R1	Interleukin 1 receptor, type I	Hs.557403
IL6	Interleukin 6 (interferon, beta 2)	Hs.512234
IL8	Interleukin 8	Hs.624
IRAK1	Interleukin-1 receptor-associated kinase 1	Hs.522819
IRAK2	Interleukin-1 receptor-associated kinase 2	Hs.449207
JUN	V-jun sarcoma virus 17 oncogene homolog (avian)	Hs.525704
LTA	Lymphotoxin alpha (TNF superfamily, member 1)	Hs.36
LTBR	Lymphotoxin beta receptor (TNFR superfamily, member 3)	Hs.1116
MALT1	Mucosa associated lymphoid tissue lymphoma translocation gene 1	Hs.180566
MAP3K1	Mitogen-activated protein kinase kinase kinase 1	Hs.508461
MYD88	Myeloid differentiation primary response gene (88)	Hs.82116
NALP12	NACHT, leucine rich repeat and PYD containing 12	Hs.367965
NFKB1	Nuclear factor of kappa light polypeptide gene enhancer in B-cells 1 (p105)	Hs.431926
NFKB2	Nuclear factor of kappa light polypeptide gene enhancer in B-cells 2 (p49/p100)	Hs.73090
NFKBIA	Nuclear factor of kappa light polypeptide gene enhancer in B-cells inhibitor, alpha	Hs.81328
PPM1A	Protein phosphatase 1A (formerly 2C), magnesium-dependent, alpha isoform	Hs.130036
RAF1	V-raf-1 murine leukemia viral oncogene homolog 1	Hs.159130
REL	V-rel reticuloendotheliosis viral oncogene homolog (avian)	Hs.370620
RELA	V-rel reticuloendotheliosis viral oncogene homolog A, nuclear	Hs.502875

	factor of kappa light polypeptide gene enhancer in B-cells 3, p65 (avian)	
RELB	V-rel reticuloendotheliosis viral oncogene homolog B, nuclear factor of kappa light polypeptide gene enhancer in B-cells 3 (avian)	Hs.307905
RFP2	Ret finger protein 2	Hs.436922
RHOA	Ras homolog gene family, member A	Hs.247077
RIPK1	Receptor (TNFRSF)-interacting serine-threonine kinase 1	Hs.519842
SLC20A1	Solute carrier family 20 (phosphate transporter), member 1	Hs.187946
STAT1	Signal transducer and activator of transcription 1, 91kDa	Hs.470943
TBK1	TANK-binding kinase 1	Hs.505874
TICAM2	Toll-like receptor adaptor molecule 2	Hs.278391
TLR1	Toll-like receptor 1	Hs.111805
TLR2	Toll-like receptor 2	Hs.519033
TLR3	Toll-like receptor 3	Hs.29499
TLR4	Toll-like receptor 4	Hs.174312
TLR6	Toll-like receptor 6	Hs.366986
TLR7	Toll-like receptor 7	Hs.443036
TLR8	Toll-like receptor 8	Hs.272410
TLR9	Toll-like receptor 9	Hs.87968
TMED4	Transmembrane emp24 protein transport domain containing 4	Hs.510745
TNF	Tumor necrosis factor (TNF superfamily, member 2)	Hs.241570
TNFAIP3	Tumor necrosis factor, alpha-induced protein 3	Hs.211600
TNFRSF10A	Tumor necrosis factor receptor superfamily, member 10a	Hs.401745

TNFRSF10B	Tumor necrosis factor receptor superfamily, member 10b	Hs.521456
TNFRSF1A	Tumor necrosis factor receptor superfamily, member 1A	Hs.279594
TNFRSF7	Tumor necrosis factor receptor superfamily, member 7	Hs.355307
TNFSF10	Tumor necrosis factor (ligand) superfamily, member 10	Hs.478275
TNFSF14[#]	Tumor necrosis factor (ligand) superfamily, member 14	Hs.129708
TRADD	TNFRSF1A-associated via death domain	Hs.460996
TICAM1	Toll-like receptor adaptor molecule 1	Hs.29344

Genes undetectable in PBMCs (Ct expression value >35)
